# RNA Polymerase I Dysfunction Underlying Craniofacial Syndromes: Integrated Genetic Analysis Reveals Parallels to 22q11.2 Deletion Syndrome

**DOI:** 10.3390/genes16091063

**Published:** 2025-09-10

**Authors:** Spencer Silvey, Scott Lovell, Merlin G. Butler

**Affiliations:** 1 Departments of Psychiatry & Behavioral Sciences and Pediatrics, University of Kansas Medical Center, Kansas City, KS 66160, USA; ssilvey@kumc.edu; 2 Protein Structure and X-Ray Crystallography Laboratory, University of Kansas, Lawrence, KS 66047, USA; swlovell@ku.edu

**Keywords:** RNA polymerase I disorders, STRING genetic mechanisms, functions, pathways, cellular components, human phenotypes, and ribosomopathies, craniofacial, cardiac, skeletal, and neurodevelopmental anomalies, protein profiling, genetic and clinical syndromic correlations

## Abstract

Background/Objective: POLR1A and related gene variants cause craniofacial and developmental syndromes, including Acrofacial Dysostosis-Cincinnati, Treacher-Collins types 2–4, and TWIST1-associated disorders. Using a patient case integrated with molecular analyses, we aimed to clarify shared pathogenic mechanisms and propose these conditions as part of a spectrum of RNA polymerase I (Pol I)–related ribosomopathies. Methods: A patient with a heterozygous POLR1A variant underwent clinical evaluation. Findings were integrated with a literature review of craniofacial syndromes to identify overlapping fea tures. Protein-protein and gene-gene interactions were analyzed with STRING and Pathway Commons, a structural modeling of POLR1A assessed the mutation’s impact. Results: The patient exhibited features overlapping with Sweeney-Cox, Saethre-Cox, Robinow-Sorauf, and Treacher-Collins types 2–4, supporting a shared spectrum. Computational analyses identified POLR1A-associated partners and pathways converging on Pol I function, ribosomal biogenesis, and nucleolar processes. Structural modeling of the Met496Ile variant suggested disruption of DNA binding and polymerase activity, linking molecular dysfunction to the clinical phenotype. Conclusion: Significant clinical and genetic overlap exists among Saethre-Chotzen, Sweeney-Cox, Treacher-Collins types 2–4, and Acrofacial Dysostosis-Cincinnati. POLR1A and related Pol I subunits provide a mechanistic basis through impaired nucleolar organization and rRNA transcription, contributing to abnormal craniofacial development. Integrative protein, gene, and structural analyses support classifying these syndromes as Pol I–related ribosomopathies, with implications for diagnosis, counseling, and future mechanistic or therapeutic studies.

## 1. Introduction

Disorders caused by POLR1A and related gene variants, including Acrofacial Dysostosis—Cincinnati type, Treacher–Collins syndrome types 2–4, and TWIST1-associated syndromes such as Saethre–Chotzen and Sweeney–Cox, exhibit overlapping craniofacial and developmental phenotypes. By combining novel clinical observations with a literature-driven evaluation of Pol I and TWIST1-associated pathways, we aim to clarify shared pathogenic mechanisms and propose a framework for considering these disorders as part of an emerging spectrum of Pol I gene-related ribosomopathies.

The identification of various clinical features overlapping in dysmorphic genetic disorders with the same related genetic causation has been reported over the years, as classically illustrated in DiGeorge syndrome, in patients presenting with cardiovascular anomalies, hypoparathyroidism with low calcium levels and cellular immune deficiency. Subsequently, Shprintzen et al. [1] reported children with velopharyngeal incompetence, cardiac defects, and a prominent nose, coined Shprintzen syndrome [2] or velo-cardio-facial disorder [3]. The patterns of malformation rapidly expanded to include defects of the third and fourth branchial arch, dysmorphic facial features with cleft palate and specific congenital heart defects. Patients presenting with these related clinical features were found to share a chromosome 22q11.2 deletion using FISH technology or high-resolution microarray analysis. Later, CATCH22, an acronym, was reported, which shared the same clinical features in patients with the chromosome 22q deletion [3]. Furthermore, Butler and Mowrey et al. [4] reanalyzed a 34-year-old male reported by Lynch et al. [5] diagnosed with 3C (cranio-cerebello-cardiac) syndrome, with cerebellar atrophy, neonatal hypocalcemia, atrial septal defect, cleft palate, a dysmorphic face, and a 22q11.2 deletion. They proposed that 3C syndrome could be included in the clinical spectrum of velo-cardio-facial, Shprintzen and DiGeorge syndromes, or CATCH22. These related genetic disorders share a 22q11.2 deletion identified independently over several years.

Common clinical findings of 22q11.2 deletion syndrome (now labeled 22q11DS) include immunodeficiency, cleft palate, laryngeal defects, congenital heart defects (some severe or lethal), hypocalcemia, craniofacial anomalies, variable eye abnormalities, and developmental or behavioral problems, including autism [3]. Additional features may include hearing loss, skeletal defects, and genitourinary abnormalities. Over 30 genes reside in the 22q11.2 region, and the recognition of multiple disorders over decades under different names has highlighted that these syndromes often share a common genetic cause: a deletion of chromosome 22q11.2 [6].

Similarly, we propose that the disorders discussed in our report are associated with disturbances of the POLR1A gene on chromosome 2, potentially involving interacting genes that generate a shared spectrum of clinical findings. Pol I-related disorders include Acrofacial Dysostosis—Cincinnati type (OMIM, 616462) [7], which shares features with Sweeney–Cox (OMIM 617746), Saethre–Chotzen (OMIM 101400), Treacher–Collins types 2–4 (OMIM 613717, 248390, and 248390), and historically, Robinow–Sorauf syndromes (OMIM, 180750). These syndromes involve overlapping RNA polymerase I disturbances, molecular functions, and biological pathways, resulting in similar clinical presentations. Here, we present a patient with Acrofacial Dysostosis—Cincinnati type as an anchor for integrative analysis of published genetic and mechanistic data. Specifically, we apply the integrative protein–protein and gene–gene analysis of POLR1A, the structural modeling of the Met496Ile variant, and comparisons across related syndromes to clarify shared pathogenic mechanisms and define an emerging spectrum of Pol I gene-related disorders.

## 2. POLR1A Gene, Protein, and Function

The POLR1A (subunit A of RNA Polymerase I) gene, located on chromosome 2p11.2, encodes a 194 kDa protein [7,8] that engages with RNA polymerase I (Pol I), a 590 kDa enzyme, consisting of 14 protein subunits. POLR1A protein is essential for Pol I function and comprises the catalytic subunit [9]. Pol I transcribes ribosomal RNA (rRNA), the most abundant RNA species in eukaryotic cells, which constitutes the catalytic core of ribosomes. rRNA acts as a ribozyme, facilitating tRNA–mRNA interactions during translation, and transcribed from ~600 rDNA repeats in the nucleolus [10,11].

The process of ribosome assembly, coined ribosome biogenesis, is a complex and delicate process involving the coordination of more than 200 proteins [12]. Ribosome biogenesis is energetically expensive, and cells can modulate this process based on nutrient availability [13]. Thus, both the hyperactivation of Pol I (as seen with Myc overexpression in cancer) and insufficient Pol I activity (due to genetic mutations causing ribosomopathies) can drive pathology [14]. Pathologies also arise when Pol I activity is insufficient, either through erroneous downregulation, or from Pol I deficiencies, due to genetic mutations. Interestingly, in human disorders, the latter is associated with a family of congenital disorders referred to as ribosomopathies. These disorders are characterized by ribosomal haploinsufficiency or defects in ribosome biogenesis and targeted protein production [15].

Animal studies highlight the essential role of POLR1A in embryonic development. In zebrafish, loss-of-function variants cause severe craniofacial and cardiac malformations, depletion of neural crest progenitors, and early embryonic lethality. Similarly, knockout mice are embryonic lethal, with conditional models revealing impaired neural crest proliferation, facial malformations, and cardiac outflow tract defects. Expression studies in both models show early ubiquitous POLR1A expression that becomes enriched in the brain, eyes, branchial arches, and frontonasal prominences—sites corresponding to tissues most affected in human POLR1A-related disorders [7,16,17].

## 3. Methods

**Case Identification**

The patient described was diagnosed and followed clinically by a board-certified geneticist. Clinical features were documented through standard genetic assessment, imaging, and laboratory investigations. Exome sequencing identified a heterozygous, likely pathogenic POLR1A variant (c.1488G > T, p.Met496Ile).

**Literature Review**

A systematic literature review was performed using PubMed, OMIM, and genetic databases to identify disorders related to POLR1A and associated genes, including Treacher–Collins, Saethre–Chotzen, Sweeney–Cox, and Acrofacial Dysostosis syndromes. Articles were screened for clinical, molecular, and structural data relevant to RNA polymerase I-related ribosomopathies. Reference lists were also checked for additional relevant publications.

**Integrated Genetic Analysis**

Protein–protein interactions involving POLR1A were analyzed using the STRING database (www.string-db.org) (accessed on 22 November 2024). Predicted and experimentally validated functional associations, network nodes, the enrichment of biological processes, molecular functions, cellular components, pathways, and human phenotype correlations were identified. Analysis of POLR1A related proteins was constructed via the analysis page, and associations were ordered by strength. Gene–gene interactions were examined via Pathway Commons and related databases to identify functional partners, co-expression patterns, and potential developmental or disease relevance.

**Structural Modeling**

The POLR1A protein structure, including the site of the patient-specific mutation (Met496Ile), was evaluated using published cryo-EM Pol I structures (PDB 7VBA and related models). Structural assessment focused on the location of the mutation relative to the DNA-binding and catalytic regions and interactions with initiation factors (e.g., RRN3).

**Data Synthesis and Analysis**

Clinical, molecular, structural, and computational data were integrated to construct a comprehensive profile of POLR1A-related disorders. Comparative tables were created to summarize overlapping clinical features across syndromes, highlight associated genes and proteins, and illustrate predicted functional interactions. Enrichment metrics, statistical significance (FDR), and pathway correlations were calculated using built-in STRING algorithms and standard bioinformatics tools.

## 4. Results

### 4.1. Table of Pol I-Associated Disorders and Case Report

#### 4.1.1. Anchoring Case

**Figure 1:**
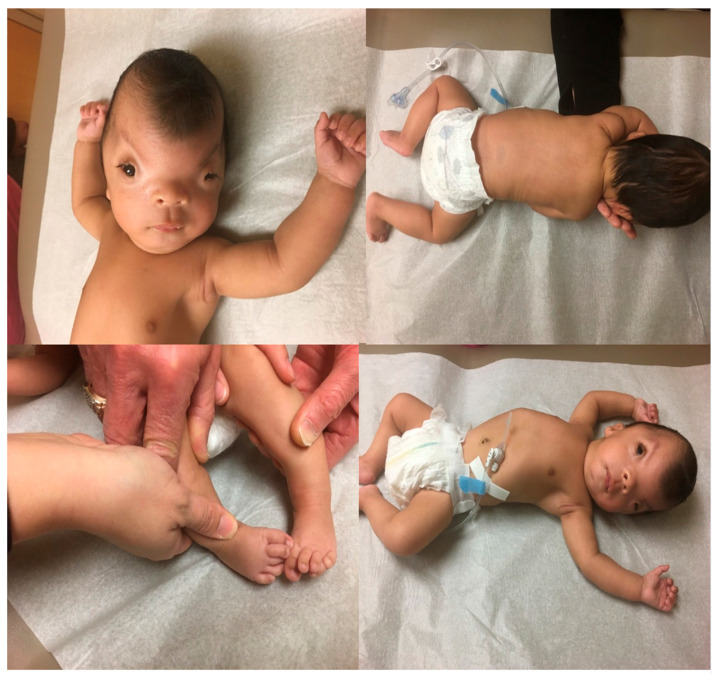
Craniofacial and full body views of front, back, and extremities of the male proband at 3 months of age. A pathogenic missense POLR1A variant was found at c.1488G > T (p.Met496Ile).

One of the authors (MGB), a clinical geneticist, diagnosed and followed the patient in the clinical setting with a de novo POLR1A gene defect. At three months of age, the patient presented for genetic services due to his constellation of cranio-facial abnormalities, skeletal deformities, acalvaria, malformed eye lids with extreme hypertelorism, small malformed ears with small canals, low hairline, a small chin, absent gag reflex, and a cardiac defect of the atrium (Figure 1). The patient was born at 36 weeks’ gestation, with a mild brain bleed during delivery. Exome testing showed a heterozygous, likely pathogenic variant of the POLR1A gene, at c.1488G > T (p. Met496Ile). His findings were clinically and genetically consistent with Acrofacial Dysostosis, Cincinnati-type, supported by a POLR1A gene defect. His clinical features overlapped with other disorders such as Sweeney–Cox, Saethre–Chotzen, Robinow–Sorauf, and Treacher–Collins syndromes and inspired our study to investigate the genetic mechanisms, biological processes, and interactions related to these rare genetic disorders. Our patient died at 4 years of age. Furthermore, our patient was reported, unbeknownst to us, with limited clinical, genetic, and protein data or analysis by Smallwood et al. [7] and was listed as patient 8 in their study.

#### 4.1.2. Table of Pol 1 Related Clinical Disorders

Upon further review of this patient, his clinical features were highly reminiscent of Sweeney–Cox, Saethre–Chotzen, and Robinow–Sorauf syndromes and Treacher Collins types 1, 2, and 3 (see Table 1). The clinical and genetic evaluation of our patient led to further investigation and studies into the POLR1A gene and its interactions with other gene pathways, related functions recognized, and related ribosomopathies disorders. A summary of potential Pol I gene-related disorders with their inheritance patterns and clinical findings of the five clinically related syndromes are described in Table 1. Terms are highlighted that appear more than once within a row, and are then included in the overlapping features tab.

### 4.2. Molecular Analysis: POLR1A Protein and Gene Interactions

We undertook a detailed study of the *POLR1A* gene and interactions with other pathways, and related functions specifically disorders involving ribosomopathies. Association with other disorders were further characterized by an investigation into *POLR1A* protein–protein interactions, gene–gene interactions, biological functions, and pathways using established computer-based programs. The POLR1A subunit was further characterized through protein modeling, with an example of the mutated protein present in our illustrative case.

#### 4.2.1. Protein–Protein Interactions and Literature Review of Associated Proteins

**Figure 2:**
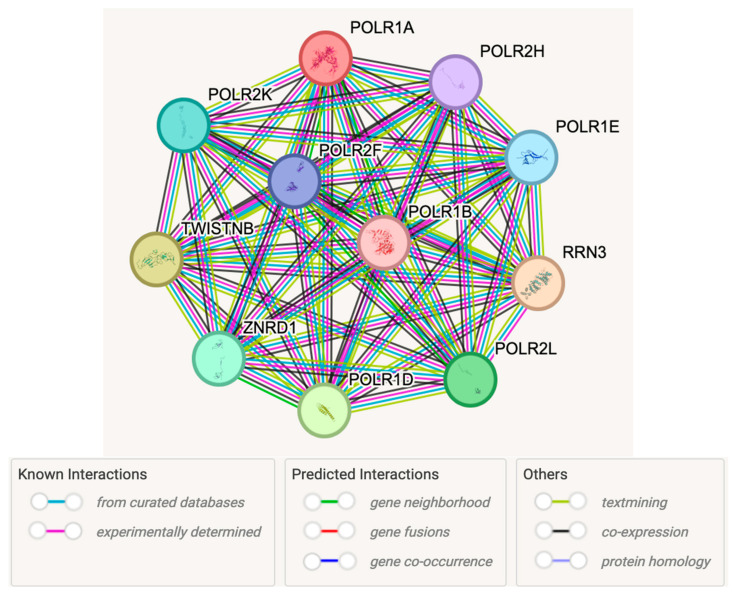
STRING protein–protein interactions with POLR1A. Associations indicate shared function and interactions among proteins. The legend and the color corresponding to the connecting lines describe whether the interaction is known, experimentally, via database, or based on predicted interactions and similarities.

Congenital anomalies in genetic disorders are caused by a combination of genetic and environmental factors that contribute to altered critical molecular pathways during embryogenesis. To further understand the role of the POLR1A gene seen in our patient, the STRING computer-based program and database (www.string-db.org) (accessed on 22 November 2024). was used to search for predicted protein–protein associations, functional interactions and enrichments, biological networks, pathways, cellular components, and human phenotypes [18]. Our study found 10 protein network nodes with each node representing proteins with splice isoforms or post-translational modifications, and then collapsed into each node, corresponding to a single protein-coding gene, such as POLR1A (Figure 2). Fifty-five edges were found, which are considered specific and meaningful, indicative of both direct and predicted functional and physical protein–protein associations with interactions that contribute jointly to a shared function. The top ten associated proteins or predicted functional partners found for POLR1A gene using the STRING database are shown with shared biological processes, molecular functions, and pathways with interactions and correlation for human phenotypes are described. These relationships are ordered by strength of association (Table 2). The related proteins identified via String analysis are further expanded upon in Table 3.

#### 4.2.2. Gene–Gene Interactions and Literature Review of Associated Genes

**Figure 3:**
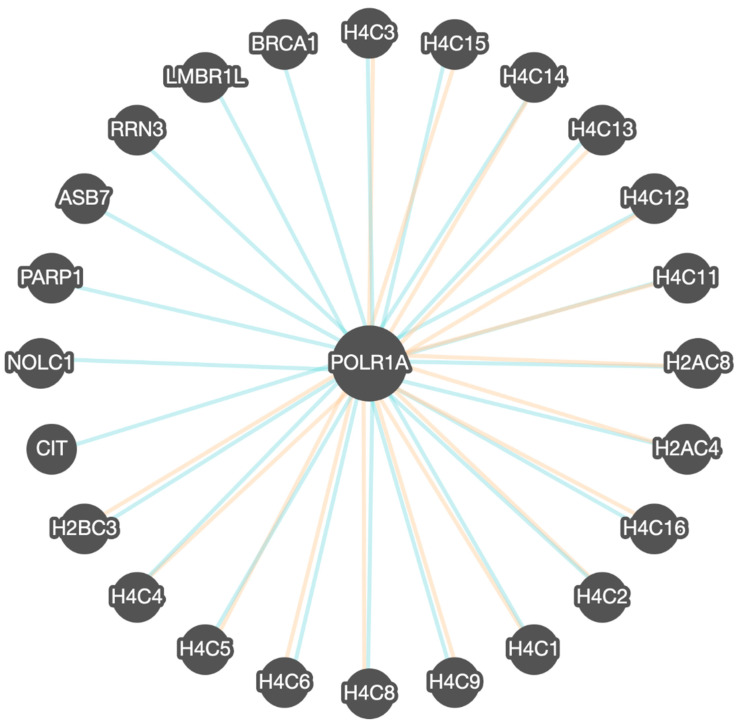
POLR1A gene–gene functional interactions determined via binding (blue lines) and co-expression (orange lines). *POLR1A* gene is highly related to the histone family of genes involved in DNA access and activity (e.g., *H4C*) and RNA transcription (e.g., *RRN3*) with nucleolar and chromatin function (e.g., *PARP1, NOLC1*), protein synthesis and degradation (*ASB7*), and developmental/congenital defects such as microcephaly (e.g., CIT, LMBR1L) and BRCA1 gene causing breast cancer (https://apps.pathwaycommons.org/search?type=Pathway&q=POLR1A) accessed on 24 August 2025.

POLR1A gene–gene interactions were divided into two categories: (1) binding partners—CIT, NOLC1, PARP1, ASB7, RRN2, LMBR1L, and BRCA1; (2) binding plus co-expression partners, primarily histone genes (Figure 3). The related genes are further expanded upon in Table 4.

#### 4.2.3. POLR1A Protein Structure and Function

**Figure 4:**
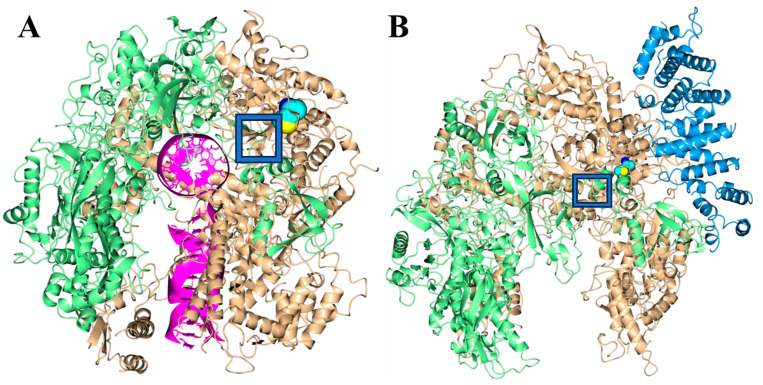
Structures of Pol I show the POLR1A (tan) and POLR1B (green) subunits. (**A**) The rendering generated from the Pol I elongation complex obtained by cryo EM (PDB 7VBA). The mixed RNA/DNA bound to the core is rendered magenta and the location of Met 496 in the POLR1A subunit is drawn as cyan spheres, drawn within a box. (**B**) The rendering generated from the Pol I structure in the complex with RRN3 (blue). The location of Met 496 in the POLR1A subunit seen in our patient is drawn as cyan spheres, drawn within a box.

Human RNA polymerase I (Pol I) is responsible for the transcription of ribosomal DNA to produce downstream RNA that is utilized for ribosome production [18]. Structurally, Pol I is a large macromolecular assembly composed of 13 subunits that have distinct roles in transcription. The POLR1A gene encodes the largest subunit (RPA1) of Pol I and contains 1720 amino acids. This RPA1 subunit along with the second largest subunit (POLR1B, RPA2) form a catalytic core that is responsible for the binding of DNA. The cryo EM structures of Pol I in the pre-translocation, post-translocation, and backtracked states have been determined which provide essential functional insight into this complex and essential enzyme [19]. The gene defects seen in our described Clinical Report of a three-month-old infant (see Figure 1) having a pathogenic missense and (c.1488G > T) change in the *POLR1A* gene at residue 496 (p.Met496Ile), as shown in Figure 4A and located near the DNA binding region. Additionally, the cryo EM structure of Pol I in the complex with the initiation factor [20] shows that the latter interacts with the region near the Met496Ile site (Figure 4B). Therefore, it is plausible that this mutation affects polymerase activity although the specific mechanism is unclear.

## 5. Review of POLR1A Gene-Related Disorder and Other Associated Ribosomopathies

### 5.1. Acrofacial Dysostosis, Cincinnati-Type

Pathogenic variants of the POLR1A gene in humans are reported to cause autosomal-dominant Acrofacial Dysostosis, Cincinnati-type, a disorder characterized by congenital craniofacial anomalies with variable limb defects. Weaver et al. [16] reported three patients with this disorder and the clinical presentation ranged from mild malar hypoplasia with dysplastic ears, micrognathia, and short, broad fingers to the severe hypoplasia of maxillary and zygomatic bones with severe micrognathia and bowed femurs. The proposed pathogenesis was due to loss of POLR1A expression, which subsequently inhibited ribosome biogenesis, thereby generating nucleolar stress and a subsequent increase in p53. An increased level of p53 may lead to neuroepithelial apoptosis, diminished neural crest cell proliferation, and observed craniofacial abnormalities characterizing the condition [16]. In contrast to other craniofacial disorders, like Treacher–Collins syndrome, which primarily causes only facial abnormalities [3]. Patients with POLR1A gene-related variants present with Acrofacial Dysostosis, Cincinnati-type, and recently shown to be associated with neural and cardiac abnormalities [7]. Furthermore, they reported patients with POLR1A gene mutations, who displayed developmental delays, infantile seizures, and congenital heart defects, most commonly atrial septal defects. Additionally, one patient with a more severe presentation, requiring surgical repair of the pulmonic and aortic artery [7].

### 5.2. Treacher–Collins Syndrome

The best-understood example of a Polymerase I-associated ribosomopathy is Treacher–Collins syndrome (TCS), a congenital disorder of craniofacial development that occurs with an estimated incidence of 1/50,000 live births [3,21]. Common clinical features of TCS include downslanting palpebral fissures, the coloboma of the eyelid, micrognathia, microtia and other ear deformity, hypoplastic zygomatic arches, and macrostomia. Conductive hearing loss and cleft palate are often present [22]. There are three reported gene mutations: a dominant *TCOF1* mutation causing TCS type 1, a recessive *POLR1B* mutation causing TCS type 2, a mixed inheritance *POLR1C* mutation causing TCS type 3, and a mixed inheritance *POLR1D* mutation causing TCS type 4. Most cases of TCS are due to a pathogenic *TCOF1* gene variant disrupting the TCOF1 protein, a vital transcription factor for the binding of rDNA promoters and Polymerase I [7]. The TCOF1 protein is also involved in the proliferation and differentiation of neural crest cells in the first and second branchial arches during embryogenesis, potentially contributing to abnormal craniofacial development seen in TCS [23,24].

### 5.3. The TWIST1 Gene and Saethre–Chotzen Syndrome

Another important gene implicated in the POLR1A gene family ribosomopathies is *TWIST*. This gene encodes a basic helix–loop–helix transcriptional regulator vital to embryonic development and is the most highly associated gene with mutations seen in Sweeney–Cox, Saethre–Chotzen, and Robinow–Sorauf syndromes. Initial studies in Drosophila demonstrated TWIST1′s key role in craniofacial and limb development via transcriptional regulation of fibroblast growth factor receptors [25]. The TWIST1 gene was cloned in humans by Bourgeois et al. [26], and its deduced 206-amino acid protein contained a DNA binding and a basic helix–loop–helix domain. Bourgeois et al. also mapped the TWIST1 gene to chromosome 7p21 [27].

Wang et al. [28] found that the TWIST1 transcript was highly expressed in the placenta with relatively lower expression in the heart and skeletal muscle with weak expression in the kidney and pancreas; however, implicated in several biological mechanisms [29]. TWIST1 also plays a role as a pro-metastatic signal in breast carcinoma via p53 inhibition [30,31] and is overexpressed in the Th1 immune cells modeling of ulcerative colitis and Crohn disease [32]. TWIST1 encodes a transcription factor critical for craniofacial development, with loss-of-function mutations causing Saethre–Chotzen syndrome, Sweeney–Cox syndrome, and Craniosynostosis 1. Saethre–Chotzen syndrome, inherited in an autosomal dominant manner, presents with premature coronal suture closure, macrocephaly, hypertelorism, midface hypoplasia, and occasionally syndactyly. Frameshift, nonsense, or missense mutations in the basic helix–loop–helix domain of TWIST1 produce truncated or dysfunctional protein, which disrupt craniofacial signaling pathways such as TWIST-FGFR2-FGFR1-CBFA1 (Runx2) [33,34,35,36,37]. TWIST1 also mutations induce osteoblast apoptosis via TNF–caspase-mediated pathways, partly mediated by TNF-alpha signaling, and impair downstream osteoblast gene expression critical for craniofacial development (e.g., FGFR2, Runx2) [38,39].

In summary, TWIST1 loss-of-function mutations lead to osteoblast apoptosis and impaired craniofacial development signaling, and in-concert, illustrate how TWIST1 mutations cause Treacher–Collins syndrome types 2, 3, and 4 and Saethre–Chotzen syndrome. Another clinical condition at one time connected with Saethre–Chotzen was the Robinow–Sorauf syndrome, also caused by a TWIST1 gene defect. It shared facial findings reminiscent of Saethre–Chotzen syndrome with the only clinical differentiator being the presence of bifid or partially duplicated halluces [40]. It is no longer classified as a separate disorder and is largely considered a phenotypic variant of Saethre–Chotzen syndrome [41].

### 5.4. Sweeney–Cox Syndrome

Sweeney–Cox syndrome (SWCOS) is another condition connected to a heterozygous TWIST1 gene mutation. SWCOS presents with a similar pattern of clinical findings to Saethre–Chotzen syndrome and Acrofacial Dysostosis, Cincinnati type, recognized in our patient, including striking facial anomalies with marked hypertelorism, prominent metopic ridge, upper eyelid colobomas, deficiency of orbital bones, mild micrognathia, cleft palate/velopharyngeal insufficiency, and cupped, low-set ears [42]. In addition, Sweeney–Cox syndrome is associated with other reported features such as a broad neck; narrow shoulders; syndactyl of the 2, 3, and 4 digits; bilateral undescended testes; an imperforate anus; hirsutism; and a low hairline. A moderate learning disability is also observed. Syndactyly was also observed along with hirsutism in unusual areas such as the back and behind knees, thickened frontal bone, and abnormal coronal and lambdoid sutures with fusion of the occipitomastoid suture. This patient was also observed to have a global developmental delay. However, while there is a paucity of data regarding Sweeney–Cox syndrome, exome sequencing in affected patients have shown a heterozygous mutation of residue 117 of the TWIST1 gene and encoded protein in a young male with a de novo E117V protein variant and in an unrelated young female with a de novo E117G protein variant [42].

## 6. Discussion

This report highlights the clinical and genetic overlap among Saethre–Chotzen, Sweeney–Cox, Treacher–Collins types 2–4, and Acrofacial Dysostosis—Cincinnati. The features of our patient initially suggested to us that there may be shared pathogenic mechanisms involving RNA polymerase I dysfunction. To explore this, we performed detailed protein–protein (Figure 2, Table 2) and gene–gene interaction (Figure 2, Table 3) analyses for POLR1A, providing novel mechanistic insights. While the precise molecular pathways remain to be fully elucidated, our findings provide a context for comparing related syndromes. Regarding Treacher–Collins, the clinical presentation and anomalies are similar to Acrofacial Dysostosis—Cincinnati, with rare eyelid colobomas, cleft palate, hypoplastic facial anomalies, conductive hearing loss, and choanal atresia [43]. Furthermore, *POLR1D* gene mutations cause Treacher–Collins syndrome, type 2, and is particularly illustrative example, as Schaefer et al. [43] demonstrated a 50% reduction in RNA transcripts with impaired RNA polymerase I activity and a subsequent reduced number of ribosomes observed in affected individuals. This suggests a similar pathogenesis to POLR1A dysfunction, further corroborated by demonstrable protein–protein interactions between the two subunits (Figure 2). Currently, data on Treacher–Collins types 3 and 4 remain limited. However, the pathogenesis of Treacher–Collins syndrome, type 4 may support the outlined hypothesis even further, as its genetic link, POLR1B, forms the other half of the Pol I dimeric catalytic subunit, along with POLR1A (Figure 4), an even clearer example of the protein–protein interrelatedness within the Pol I complex.

The overlap between Pol I-related syndromes (e.g., Treacher–Collins and Acrofacial Dysostosis—Cincinnati) and TWIST1-associated disorders (Saethre–Chotzen and Sweeney–Cox) is perhaps more complex. TWISTNB (TWIST nearby protein), also known as POLR1F, encodes a subunit of RNA polymerase I and lies approximately 580 kb upstream of TWIST1 [44]. It is associated, with POLR1A, through protein–protein interactions, and is listed as TWISTNB in Figure 2. POLR1F is expressed in all fetal and human tissues tested and resides in the same cytogenetic location (7p21.1) as TWIST1 [45]. These observations suggest that phenotypic similarities between syndromes related to RNA polymerase I and TWIST1 may depend on whether deletions at 7p21.2 encompass both TWISTNB/POLR1F and TWIST1 together or only TWIST1. A subset of patients with clinical features of Saethre–Chotzen or Sweeney–Cox syndromes (Table 1) could plausibly have RNA polymerase I disturbances via POLR1F abnormalities and may represent part of an emerging spectrum of Pol I gene-related disorders. Patients with TWIST1-only mutations are less likely to fall within this spectrum. Johnson et al. [46] identified a microdeletion at 7p21.1 encompassing TWISTNB in patients with Saethre–Chotzen syndrome, who exhibited developmental delays uncommon in patients with intragenic TWIST1 mutations. This reinforces a potential mechanistic role for POLR1F in shared molecular pathogenesis across these disorders. This observation is consistent with our integrative protein–protein and gene–gene interaction analyses, which suggest that POLR1F abnormalities may contribute to the phenotypic spectrum observed in our patient.

Our combined analysis of protein–protein and gene–gene interactions provides compelling evidence that POLR1A dysfunction disrupts key cellular pathways critical for nucleolar organization, rRNA transcription, and RNA polymerase I complex assembly (Table 2 Based on the protein–protein and gene–gene interaction networks summarized in Table 2, Table 3 and Table 4, downstream consequences on neural crest cell proliferation and craniofacial morphogenesis are predicted, consistent with the clinical phenotypes observed in Acrofacial Dysostosis—Cincinnati and related syndromes. The convergence of co-expression and binding partners, including histone genes, RRN3, and NOLC1, underscores the intersection of Pol I activity with chromatin remodeling and DNA accessibility, likely influencing developmental gene networks and contributing to overlapping craniofacial, skeletal, and systemic features (Table 1, Figure 3, and Table 4). Structurally, the Met496Ile variant identified in our patient localizes near both the DNA-binding interface and the RRN3-interacting domain of POLR1A, further suggesting a mechanistic link to impaired transcriptional initiation and Pol I complex assembly (Figure 4). While direct functional studies are required to definitively characterize its impact, the combination of computational modeling, protein interaction networks, and clinical phenotyping strengthens the hypothesis that RNA polymerase I perturbation underlies the observed developmental anomalies.

Beyond the individual variant, our integrative approach highlights the utility of combining exome sequencing with protein structural modeling and network-based bioinformatic analyses. Such strategies not only identify pathogenic variants but also predict their functional consequences within complex molecular networks. By revealing shared pathways between POLR1A- and TWIST1-associated disorders, this study provides a unifying framework for understanding ribosomopathy-driven craniofacial syndromes.

Clinically, recognizing these common molecular mechanisms may inform anticipatory management, including monitoring for skeletal, neurological, and hematological manifestations. Furthermore, elucidating the downstream signaling consequences of Pol I dysfunction opens avenues for therapeutic exploration, potentially targeting ribosome biogenesis, chromatin accessibility, or compensatory developmental pathways. Overall, these findings demonstrate the power of integrative genomics and systems biology in bridging genotype–phenotype gaps in rare developmental disorders, providing both mechanistic insight and a roadmap for translational application.

## 7. Conclusions

Our analysis highlights considerable clinical and genetic overlap among Saethre–Chotzen, Sweeney–Cox, Treacher–Collins types 2–4, and Acrofacial Dysostosis—Cincinnati, with accumulating evidence that RNA polymerase I dysfunction may represent a shared pathogenic mechanism. These findings suggest that such disorders could be viewed as part of an emerging spectrum of Pol I gene-related disorders, rather than entirely distinct entities. Recognizing these connections may have future implications for genetic testing strategies, counseling, and clinical management, particularly as disorder-specific panels expand. Our case contributes to this developing framework and underscores the importance of further studies to clarify the molecular mechanisms, genotype–phenotype correlations, and potential diagnostic utility of considering these disorders together.

## Figures and Tables

**Table 1:** Clinical and genetic findings from overlapping syndromes with POLR1A and related gene mutations. Highlights denote shared genetic or clinical presentations of two or more across each row.

	Acrofacial-Dysostosis, Cincinnati Type (616462)	Saethre–Chotzen (101400)	Sweeney–Cox (617746)	Robinow–Sorauf (180750)	Treacher–Collins 2,3, and 4 (613717, 248390, and 248390)	Clinical Report at 3 Months of Age	Overlapping Clinical Findings
Inheritance	-Autosomal Dominant	-Autosomal Dominant	-Autosomal Dominant	-Autosomal Dominant	-Autosomal Dominant	-Autosomal Dominant	-Autosomal Dominant
Growth	-Short stature -Low weight -Failure to thrive	-Short stature				-Short stature -Low weight -Failure to thrive	-Short stature -Low weight and failure to thrive
Head	-Microcephaly -Trigonocephaly	-Brachycephaly -Acrocephaly	-Flattened occiput -Brachycephaly			-Cranial deformity	-Mis-shaped head -Cranial deformity
Neck			-Wide neck				
Face	-Mild to severe midface hypoplasia -Mild to severe micrognathia -Hypoplastic zygomatic arches -Hypoplastic maxilla -Hypoplastic Mandible -Absent mandibular rami	-Flat face -High, flat forehead -Low frontal hairline -Maxillary hypoplasia -Facial asymmetry	-Prominent metopic ridge -Low hairline -Widow’s peak -Midface hypoplasia -Micrognathia, mild		-Zygomatic complex hypoplasia -Mandibular hypoplasia -Malar hypoplasia -Scalp hair onto lateral cheek	-Facial anomaly -Low hairline -Micrognathia	-Abnormal, low frontal or scalp hairline -Facial bone hypoplasia -Jawbone defect -micrognathia
Ears	-Large ears -Microtia -Thickened helices -Low-set ears -Anteriorly placed ears -Anotia with conductive hearing loss -Sensorineural hearing loss -Preauricular pits	-Long, prominent ear crus -Small ears -Low-set ears -Atpical cartilage deformity -Deafness	-Small ears -Low-set ears -Cupped ears -Overfolded helices -Upturned lobes -Narrow external ear canals -Hearing loss, bilateral		-Malformation of auricle -Microtia -Conductive hearing loss	-Small, malformed ears -Small ear canals -Low-set ears -Hearing loss	-Low-set, abnormal size, and malformed ears -Hearing loss/deafness -Abnormal ear canals
Eyes	-Downslanting palpebral fissures -Upslanting palpebral fissures -Epicanthal folds -Mild to severe hypertelorism -Telecanthus -Eyelid clefts -Eyelid coloboma -Ablepharon -Ptosis -Inferiorly displaced orbits	-Shallow orbits -Hypertelorism -Plagiocephaly -Strabismus -Bupthalmos -Ptosis -S-shaped blepharoptosis -Lacrimal Duct abnormalities	-Hypertelorism -Upper eyelid colobomas -Deficient bony orbits -Pseudoproptosis -Small globes	-Shallow orbits -Hypertelorism -Plagiocephaly -Strabismus	-Downslanting of palpebral fissure -Coloboma	-Severe hypertelorism -Coloboma -Right eye Telecanthus	-Telecanthus -Colobomas -Hypertelorism -Orbital defects -Strabismus -Slanted palpebral fissures -Ptosis
Nose	-Broad, flat nasal bridge -Short nose -Upturned nasal tip -Anteverted nares -Hypoplastic alae nasi -Choanal atresia	-Thin, long pointed nose -Beaked nose	-Wide nasal bridge -Broad tip -Hypoplastic alae nasi -Choanal atresia -Short and low columella -Short philtrum	-Long, pointed nose	-Choanal atresia/stenosis		-Abnormal hypoplastic alae -Choanal anomalies
Mouth	-High-arched palate -Cleft palate	-Narrow palate -Cleft palate	-Small mouth -Cleft-palate -High-arched palate		-Cleft palate		-Abnormal palate with cleft
Cardiovascular	-Patent ductus arteriosus -Atrial septal defect -Patent foramen ovale -Hypertrophic cardiomyopathy	-Congenital defects -Hypertension				-Atrial cardiac defect	-Cardiac defects
Skull	-Microcephaly -Metopic craniosynostosis -Partial acalvaria	-Late closing fontanelles -Craniosynostosis of coronal, lamboid, or metopic structures -Acrocephaly -Parietal foramina	-Small frontal bones -Thickened frontal bones -Wide anterior fontanel -Fusion of occipito-mastoid suture			-Acalvaria -Craniosynostosis	-Skull defects -Craniosynostosis
Abdomen/GU/Pelvis	-Dysplastic acetabulae -Feeding problems	-Small Ilia -Small ischia	-GERD -Imperforate anus -Absent spleen -Cryptorchidism			-Cryptorchidism -Gastric tube placement	-Cryptorchidism -GI defects
Chest			-Short clavicle -Convex medial margins of scapulae				
Limbs	-Short, bowed forearms -Radial aplasia -Bowed femurs -Flared metaphyses of lower extremities -Delayed ossification of epiphyses	-Radioulnar synostosis					
Hands	-Short broad digits -Preaxial polydactyly -Adducted thumb with bifid distal phalanx -Fifth finger clinodactyly and short medial phalanx	-Syndactyly, mild and often in second or third fingers -Bifid terminal phalanges -Brachydactyly -Fifth finger clinodactyly	-Long fingers -Relatively short and fixed flexion of distal phalanges -Cutaneous syndactyly				-Syndactyly -Digital anomalies
Feet	-Short toes -Triphalangeal halluces -Overriding toes -Club feet	-Absent first metatarsal -Syndactyly -Hallux vagus	-Cutaneous syndactyly	-Broad great toes -Duplicated distal toe phalanx			-Syndactyly -Digital anomalies
Neurologic	-Hypotonia -Global developmental delay -Motor delay -Delayed or absent speech -Infantile spasms -Epilepsy -Cavum septum pellicidum	-Global developmental delay	-Developmental and learning disability -Speech delay -Small cerebellum -Hypoplastic facial nerves		-Motor delay -Speech delay		-Global developmental delay -Speech and motor delay -CNS anomalies
Other	-Asymmetric thumb nails	-Increased risk of breast cancer	-Generalized hirsutism -Abnormal hair distribution on back and ankles				
Molecular genetics	-POLR1A	-TWIST1	-TWIST1	-TWIST1	-POLR1D (2) -POLR1C (3) -POLR1B (4)	-POLR1A	-POLR1A -TWIST1

**Table 2:** Analysis and results of the ten interactive proteins associated with POLR1A identified using STRING computer-based program with shared and corresponding biological processes (Gene Ontology), molecular functions (Gene Ontology), cellular components (Gene Ontology), pathways (Reactome), and human phenotype correlations.

Biological Process	CIN ^A^	Strength ^B^	Signal ^C^	FDR ^D^
Transcription by RNA polymerase I	5 of 25	2.55	4.37	3.76 × 10^−9^
RNA polymerase I preinitiation complex assembly	2 of 10	2.55	1.08	0.0108
Nucleolus organization	2 of 15	2.38	0.93	0.0194
rRNA transcription	2 of 21	2.23	0.81	0.0328
ncRNA transcription	3 of 38	2.15	1.57	0.00097
DNA-templated transcription, initiation	3 of 131	1.61	0.8	0.0239
Transcription, DNA-templated	11 of 518	1.58	3.44	7.37 × 10^−14^
Transcription by RNA polymerase II	4 of 357	1.3	0.76	0.0172
Molecular Function	CIN	Strength	Signal	FDR
DNA-directed 5-3 RNA polymerase activity	6 of 29	2.57	5.29	4.52 × 10^−11^
DNA binding	9 of 2498	0.81	0.71	0.00026
Nucleic acid binding	10 of 4003	0.65	0.53	0.00067
Cellular Component	CIN	Strength	Signal	FDR
RNA polymerase I complex	10 of 13	3.14	13.65	1.06 × 10^−26^
RNA polymerase III complex	5 of 19	2.67	5.09	1.79 × 10^−10^
RNA polymerase II, core complex	4 of 15	2.68	3.96	3.46 × 10^−18^
Nucleolus	11 of 996	1.3	2.22	2.01 × 10^−12^
Nucleoplasm	11 of 4169	0.67	0.63	6.08 × 10^−6^
Fibrillar center	3 of 145	1.57	1.04	0.0058
Reactome Pathway	CIN	Strength	Signal	FDR
RNA polymerase I transcription termination	9 of 30	2.73	10.01	7.16 × 10^−21^
RNA polymerase III chain elongation	4 of 17	2.62	3.82	5.95 × 10^−8^
RNA polymerase I transcription initiation	10 of 46	2.59	10.13	3.67 × 10^−22^
Signaling by FGFR2 IIIa	4 of 19	2.58	3.73	8.12 × 10^−8^
RNA Polymerase III transcription termination	4 of 22	2.51	3.6	1.27 × 10^−7^
Abortive elongation of HIV-1 transcript in absence of Tat	4 of 23	2.49	3.57	1.39 × 10^−7^
RNA Polymerase I promoter escape	10 of 59	2.48	9.46	1.75 × 10^−21^
MicroRNA (miRNA) biogenesis	4 of 24	2.47	3.54	1.52 × 10^−7^
B-WICH complex positively regulates rRNA expression	9 of 59	2.44	8.29	8.31 × 10^−19^
RNA Polymerase III transcription initiation from type 2 promoter	4 of 26	2.44	3.47	1.92 × 10^−7^
FGFR2 alternative splicing	4 of 26	2.44	3.47	1.92 × 10^−7^
Phenotype	CIN	Strength	Signal	FDR
Branchial fistula	2 of 6	2.78	0.99	0.0161
Lower eyelid coloboma	2 of 8	2.65	0.94	0.0194
Multiple enchondromatosis	2 of 9	2.6	0.92	0.0211
Choanal stenosis	3 of 21	2.41	1.35	0.0032
Narrow internal auditory canal	2 of 14	2.41	0.84	0.0297
Short face	2 of 16	2.35	0.81	0.0329
Facial cleft	2 of 16	2.35	0.81	0.0329
Eyelid coloboma	3 of 30	2.25	1.27	0.0043
Rectovaginal fistula	2 of 20	2.25	0.79	0.0356
Thyroid hypoplasia	2 of 21	2.23	0.79	0.0356
Absent eyelashes	2 of 26	2.14	0.73	0.0447
Glossoptosis	2 of 26	2.14	0.73	0.0447
Hypoplasia of the zygomatic bone	2 of 29	2.09	0.71	0.0477
Hypoplasia of the thymus	2 of 30	2.08	0.71	0.0477
Blepharospasm	2 of 32	2.05	0.70	0.0492
Preauricular skin tag	3 of 51	2.02	1.06	0.0096
Abnormal periauricular region morphology	3 of 77	1.84	0.90	0.0181
Choanal atresia	3 of 101	1.73	0.79	0.0279
Hypoplasia of the maxilla	3 of 104	1.71	0.79	0.0279
Microtia	3 of 113	1.68	0.77	0.0297
Abnormality of the maxilla	3 of 118	1.66	0.77	0.0297
Cleft upper lip	3 of 144	1.57	0.72	0.0356
Cleft lip	3 of 183	1.47	0.64	0.0477
Narrow mouth	3 of 198	1.43	0.63	0.0495

^A^ CIN (count-in-network) indicates how many proteins in network are annotated with a particular term and how many proteins in total (in network and in the background) have this term assigned to this variable per category (Biological Process, Molecular Function, etc.). ^B^ Log10 (observed/expected) or strength. This measure describes how large the enrichment effect is with the ratio between (i) the number of proteins in network that are annotated with a term and (ii) the number of proteins expected to be annotated with this term in a random network of the same size. ^C^ Signal is defined as a weighted harmonic mean between the observed/expected ratio and −log(FDR). FDR or false discovery rate tends to emphasize larger terms due to their potential for achieving lower *p*-values, while the observed/expected ratio highlights smaller terms, which have a high foreground to background ratio but cannot achieve low FDR values due to their size. ^D^ FDR is a statistical measure that describes the significance of enrichment. Shown are *p*-values corrected for multiple testing within each category.

**Table 3:** RNA Polymerase1A-associated proteins, functions, and disorders.

Gene	Protein Function/Role ^1^	Pathways/Interactions ^2^	Associated Disorders ^3^
POLR1F (TWISTNB)	Subunit of RNA polymerase I; enables DNA-directed 5′–3′ RNA polymerase activity; essential for rRNA precursor synthesis and protein production.	Associates with RRN3/TIF-IA; may recruit Pol I to rDNA promoters.	Inflammatory bowel disease 2; Saethre–Chotzen syndrome
RRN3	RNA Pol I transcription factor; enables core promoter sequence-specific DNA binding; regulates transcription initiation; negative regulation of p53-mediated apoptosis.	Associates with POLR1A; nucleolar localization.	Childhood-onset neurodegeneration with brain atrophy; Treacher–Collins syndrome 1 (potential)
POLR1D	Component of RNA polymerase I and III complexes; synthesizes rRNA precursors and small RNAs.	Core Pol I/Pol III subunit.	Treacher–Collins syndrome 1 and 2
POLR2L	Subunit of RNA polymerase II; synthesizes mRNA; contains zinc-binding domain.	RNA Pol II complex.	Cockayne syndrome; Hyperparathyroidism 2 with jaw tumors
POLR1H	Subunit of RNA polymerase I; contains zinc-binding motifs; involved in proofreading nascent RNA and regulation of cell proliferation.	Functions in Pol I error correction.	Gastric cancer; Seckel syndrome
POLR2K	Small subunit of RNA polymerase II; involved in mRNA synthesis.	RNA Pol II complex.	Immunodeficiency 26; Primary ciliary dyskinesia
POLR1E	Facilitates Pol I initiation complex formation by mediating Pol I–UBTF interaction; contributes to DNA binding and Pol I transcription.	Pol I initiation factor binding.	Primary hyperoxaluria; Diamond–Blackfan anemia
POLR2F	Sixth largest Pol II subunit; mRNA synthesis.	RNA Pol II complex.	Peripheral demyelinating neuropathy, central dysmyelination, Waardenburg syndrome, Hirschsprung disease
POLR2H	Essential Pol II subunit; contributes to Pol I and II activity; synthesizes rRNA, mRNA, small RNAs (e.g., 5S rRNA, tRNAs).	Shared between Pol I and Pol II.	Progressive leukoencephalopathy with ovarian failure
POLR1B	Core catalytic subunit of RNA Pol I active center; works with POLR1A in nucleotide addition, proofreading, and backtracking.	Coordinates Mg^2+^ with POLR1A; proofreading via POLR1H.	Treacher–Collins syndrome 1 and 4

^1^ Molecular functions for POLR1A subunits were obtained from STRING (www.string-db.org (accessed on 24 August 2025)). ^2^ Pathways for associated POLR1A subunits were obtained from Gene Ontology (GO) (November 2024). ^3^ Associated human disorders were retrieved from OMIM (November 2024).

**Table 4:** RNA Polymerase 1A-associated genes, with functions and associated disorders.

Category	Gene	Function/Role ^1^	Associated Disorders ^2^
Binding	CIT	Serine/threonine kinase important for cytokinesis; works with KIF14 at central spindle.	Bipolar disorder, schizophrenia
Binding	NOLC1	Nucleolar protein connecting RNA Pol I to ribosomal processing enzymes; monoubiquitinated by BCR complex and associates with TCOF1; essential for neural crest specification.	Neural crest development
Binding	PARP1	Poly(ADP-ribosyl) transferase; DNA-dependent chromatin enzyme regulating proliferation, differentiation, tumor suppression, and DNA repair.	Fanconi anemia, type 1 diabetes mellitus
Binding	ASB7	Ankyrin repeat protein with SOCS-box motif; bridges substrate-binding and E3 ubiquitin ligases; regulates protein turnover via ubiquitination.	
Binding	RRN3	RNA Pol I core promoter factor; initiates transcription (described in Table 1).	Childhood-onset neurodegeneration, Treacher–Collins syndrome 1 (potential)
Binding	LMBR1L	Transmembrane protein; involved in receptor-mediated endocytosis and signal transduction; localizes to ER and plasma membrane.	
Binding	BRCA1	Nuclear phosphoprotein; tumor suppressor; forms BASC complex with Pol II; regulates transcription, DNA repair, and recombination.	Hereditary breast and ovarian cancer
Binding + Co-expression	Histone genes (H2A, H2B, H3, H4, H1)	Core nucleosome proteins; package DNA into chromatin; H1 compacts DNA into higher-order structures.	

^1^ Functional annotations and molecular roles were obtained from Gene Ontology (GO) (November 2024). ^2^ Associated human disorders were retrieved from OMIM (November 2024).

## Data Availability

All data are shown in this report.
